# Tamoxifen-Dependent, Inducible Bmp2CreER Drives Selective Recombinase Activity in Early Interdigital Mesenchyme and Digit Collateral Ligaments

**DOI:** 10.1371/journal.pone.0123325

**Published:** 2015-04-07

**Authors:** Bau-Lin Huang, Susan Mackem

**Affiliations:** Cancer & Developmental Biology Laboratory, Center for Cancer Research, NCI-Frederick, Frederick, Maryland, United States of America; Laboratoire de Biologie du Développement de Villefranche-sur-Mer, FRANCE

## Abstract

During limb development, the interdigital mesenchyme has been proposed to play a signaling role instructing morphogenesis of different digit types, as well as undergoing programmed cell death necessary to free digits in animals not adapted for swimming or flying. We have generated a conditional, tamoxifen-dependent Cre line, Bmp2CreER, which drives highly selective recombination restricted to the distal limb mesoderm, largely restricted to the interdigits, and selectively active in digit ligament but not tendon progenitors at later stages. The Bmp2CreER provides a valuable new tool to dissect roles of interdigital mesenchyme and potentially investigate divergence of ligament and tendon lineages.

## Introduction

Organogenesis often entails inductive interactions involving several cell and/or tissue types and employing multiple signaling pathways to regulate cell fate, patterning and growth. Unraveling the roles of individual components in such an elaborate array of interactions poses a challenge for traditional genetics. The broad application of tamoxifen-inducible Cre-LoxP technology for the analysis of genetically engineered mice has provided a tool for selective spatial and temporal manipulation of gene function, as well as genetic lineage tracing analyses [1], to overcome such hurdles. In the developing limb, a premiere model for studying morphogenesis of a complex structure, great strides have been made in understanding how regulatory networks orchestrate this process with the use of genetic engineering strategies in mice [2, 3].

The regulation of early limb bud outgrowth and patterning has been the subject of intense study and is governed by two major signaling centers. FGFs produced by the apical ectodermal ridge (AER) instruct limb outgrowth [4], and SHH (Sonic Hedgehog) produced by the posterior marginal mesenchyme regulates antero-posterior patterning of limb elements [2, 5]. However, regulatory inputs guiding morphogenesis during the transition between early limb bud patterning and late stages of skeletal differentiation remain less well understood. The interdigital mesenchyme has been proposed to play several roles during this phase, but definitive genetic analysis has been hampered by limited tools for selectively targeting this tissue. Grafting experiments in chick demonstrated an instructive role of the interdigital mesenchyme in the late regulation of digit ‘identity’ [6], but the nature of the signals involved remains controversial and genetic evidence supporting such a role is lacking [7–9]. Shortly after the appearance of digit rays, a program of cell death is initiated in the interdigital tissue to produce free digits, in which the BMP pathway plays a central role [10, 11]. However, genetic analysis thus far indicates that mesenchymal Bmps act indirectly by promoting AER regression [12]. Whether Bmp signaling directly to the mesoderm also plays a role in directing interdigital apoptosis remains unclear.

To effectively use the large resource of conditionally targeted mouse alleles available for investigating potential interdigit roles, a tissue-specific Cre line would be highly desirable. A previously generated Hoxb6CreER line is strongly expressed in digit progenitors and gradually transitions to expression in interdigital mesenchyme; expression overlap making it difficult to select tamoxifen timing so that activity is limited entirely to interdigits [13]. An Osr1Cre transgenic line expressing Cre recombinase selectively in interdigital mesenchyme was previously generated [14], but robust recombination is not seen until about E13.5, when the interdigits have already begun to undergo regression. Several Bmps, including Bmp2, are expressed strongly in early interdigital mesenchyme and the regulatory sequences necessary for this expression have been characterized in transgenic mice [15, 16]. We used this regulatory domain to generate a transgenic line that will drive Cre activity selectively in interdigital mesenchyme. Since early stage expression may include mesenchymal cells that will give rise to both digit precursors and interdigits, a tamoxifen-inducible CreER line was generated and is described in this report.

## Results and Discussion

A conditional CreER-T line was generated containing a previously characterized 4.5kbp limb-specific enhancer located about 110kbp 3’ of the Bmp2 coding sequence [15] and the basal Hsp68 promoter. Two transgenic lines were generated from independent founders that had Cre expression in embryonic limb buds (out of 3 founders total). Preliminary characterization indicated that expression was qualitatively very similar in both lines (data not shown). Therefore, the line with a stronger expression level was used for all subsequent analyses and referred to as Bmp2CreER.

**Fig 1:**
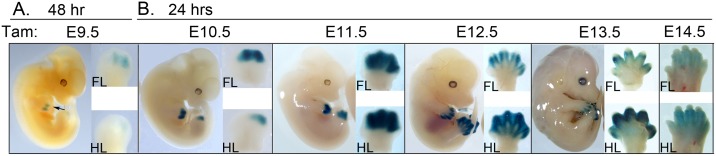
Bmp2CreER activity 24 hours after tamoxifen (Tam) treatment surveyed at daily intervals during the course of limb development. A. LacZ activity at 48 hours after tamoxifen treatment at E9.5 with arrow pointing to weak activation in distal forelimb (FL inset). Only trace expression is seen in hindlimb (HL inset). (No appreciable LacZ staining was observed at 24 hours after tamoxifen treatment; data not shown). B. LacZ activity assayed at 24 hrs after tamoxifen treatment at times indicated above each panel. All insets to the right show forelimb (FL) and hindlimb (HL) buds oriented with distal at top, and anterior-to-posterior (digit 1–5) from left-to-right. Note that no LacZ staining is evident in tissues outside of the limb.

Tamoxifen treatment prior to the time of limb initiation (at E6.5 or E7.5) produced no apparent LacZ reporter expression in any embryonic tissues assayed at E9.5 (data not shown). Appreciable Cre activity was first detectable following tamoxifen treatment at E9.5 (Fig 1A, and not shown) with LacZ reporter expression becoming evident in the distal forelimb 48 hours after treatment, suggesting that activity initiating during this period was weak, resulting in very mosaic recombination. Embryos analyzed sequentially at 24 hours after tamoxifen injection (Fig 1B) showed expression exclusively in the limb bud, as previously reported. The peak of inducible Cre activity within 24 hours of treatment was observed between E11.5 to E12.5. At later tamoxifen treatment times, LacZ reporter expression began to decline and was only weakly expressed by E14.5. Bmp2CreER was active in a restricted time window from E9.5 to E14.5 and was limited to distal limb buds (autopod), largely excluding digit one in the forelimb, but gradually extending to the posterior half of digit one in hindlimb. Expression was otherwise very similar between fore- and hindlimb, but shifted in the hindlimb stage owing to the 12-hour developmental delay relative to forelimb. For simplicity, forelimb stages are referred to here when describing Cre activity, unless otherwise indicated.

**Fig 2:**
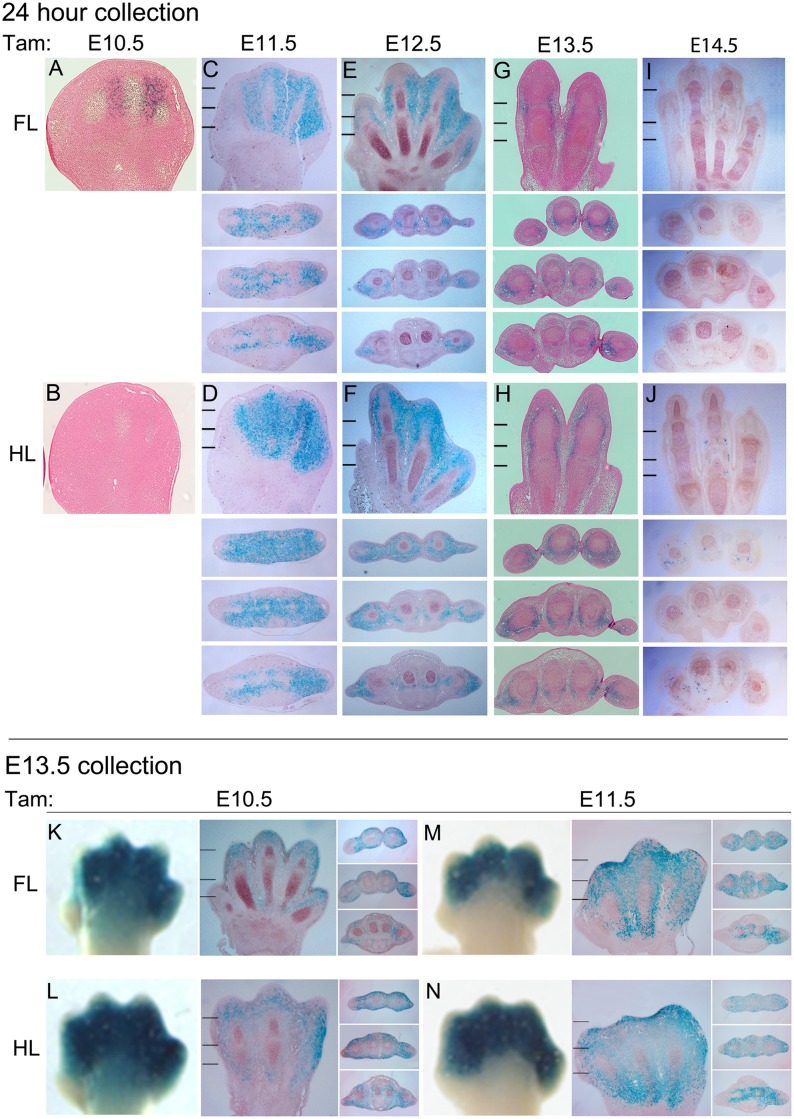
Tissue localization of Bmp2CreER activity during the course of limb development. Upper panels: 24-hour collection (A-J). Tissue localization of Bmp2CreER activity at 24 hours after tamoxifen (Tam) treatment on consecutive days from E10.5–14.5. Panels A,C,E,G,I (forelimb, FL) and B,D,F,H,J (hindlimb, HL) show longitudinal sections through limb dorsoventral axis oriented with distal at top, and anterior-to-posterior (digit 1–5) from left-to-right. Hatch marks in panels C-J show the levels at which cross-sections below panels C-J were taken (distal to proximal). Cross-sections are all oriented with dorsal at top and anterior-to-posterior (digit 1–5) from left-to-right. Lower panels: E13.5 collection (K-N). Tissue distribution of LacZ activity at E13.5 after tamoxifen (Tam) treatment at E10.5 (K,L) or E11.5 (M,N). For each treatment time, whole mount and adjacent longitudinal and cross-sections of forelimb (FL) or hindlimb (HL) are shown. Hatch marks in panels of longitudinal sections show the levels at which cross-sections through digit region were taken (distal to proximal). Longitudinal and cross-sections are oriented as for Upper Panels. Whole mount images are oriented the same as longitudinal sections.

Analysis of stained sections at 24 hours after E10.5 tamoxifen treatment (Fig 2A) revealed weak, mosaic LacZ reporter activity in the digit condensations as well as adjacent interdigital mesenchyme. After E10.5, LacZ reporter activation shifted to predominantly interdigital mesenchyme, with more uniform activation by E11.5 and declining activity after E12.5. Since Cre activity appeared to peak around E11.5 we also examined the extent of LacZ reporter activation assayed at E13.5, following tamoxifen treatment at E10.5–11.5 (Fig 2B), which confirmed activity throughout most of the interdigital mesenchyme in the distal digital rays and the sub-epidermal area of the more proximal metapodial region, excluding ventral palmar and plantar tissues (muscle and ventral tendon progenitors). A small amount of mosaic LacZ staining was also seen scattered within digital condensations.

**Fig 3:**
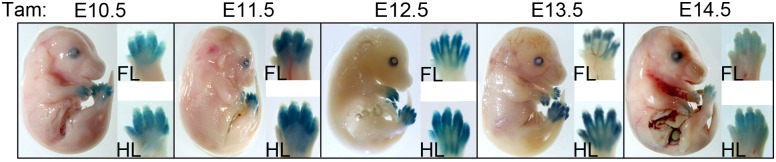
Whole mount staining of LacZ-positive descendants at E15.5 following tamoxifen (Tam) treatment at different stages from E10.5—E14.5. LacZ activity was assayed at E15.5, following tamoxifen treatment at time indicated above each panel. All insets to the right show forelimb (FL) and hindlimb (HL) buds oriented with distal at top, and anterior-to-posterior (digit 1–5) from left-to-right. Note that no LacZ staining is evident in tissues outside of the limb.

**Fig 4:**
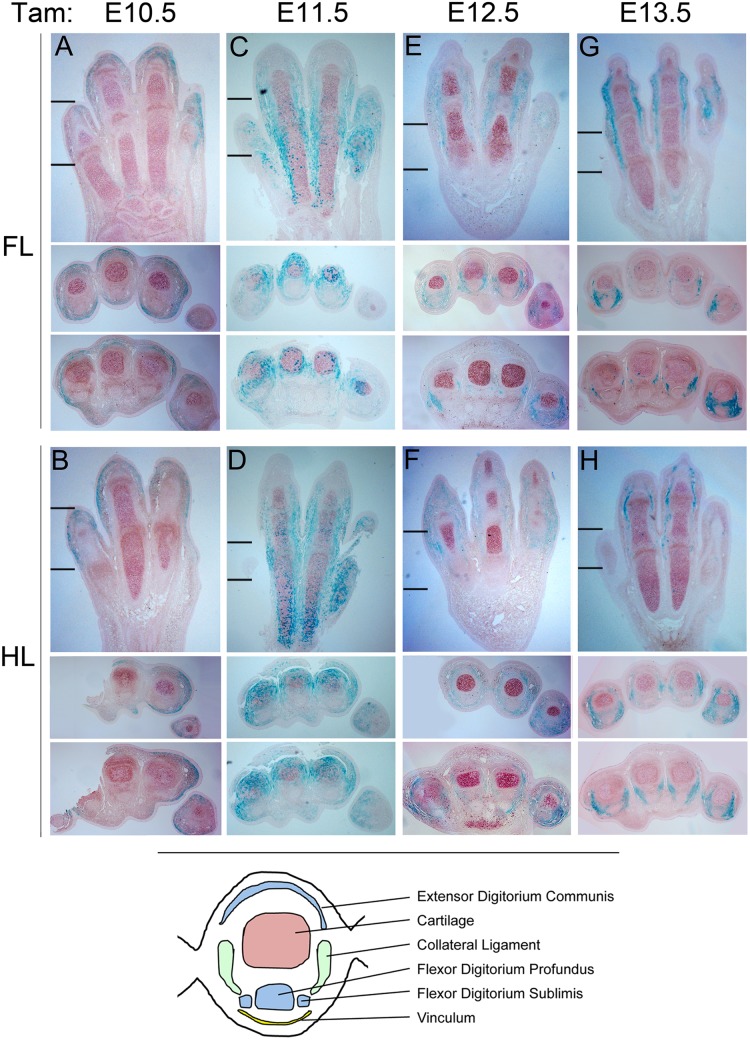
Tissue distribution of LacZ activity at E15.5 following tamoxifen (Tam) treatment at different stages from E10.5—E14.5. Panels A,C,E,G (forelimb, FL) and B,D,F,H (hindlimb, HL) show longitudinal sections through limb dorsoventral axis oriented with distal at top, and anterior-to-posterior (digit 1–5) from left-to-right. Hatch marks in panels A-H show the levels at which cross-sections below panels A-H were taken (distal, proximal). Cross-sections are all oriented with dorsal at top and anterior-to-posterior (digit 1–5) from left-to-right. Note that tamoxifen treatment at E13.5 results in LacZ staining restricted to collateral ligament. Lower panel cartoon shows a diagram of an E15.5 digit cross-section with cartilage (red), tendon (blue) and ligament (green) structures annotated for reference.

To gain a better understanding of what limb structures the cells exhibiting Cre activity contribute to at later stages, LacZ staining was also evaluated at a late time point (E15.5) following a single dose of tamoxifen given at different stages during limb development ranging from E10.5—E14.5 (Figs 3 and 4). Tamoxifen treatment at E10.5 resulted in LacZ positive descendants predominantly in superficial connective tissue underlying the epidermis in the digital region both dorsally and ventrally (Fig 4). Treatment at E11.5 resulted in LacZ activity in mesenchymal derivatives surrounding the digits, with more extensive staining dorsally, and some mosaic staining in superficial layers of condensing cartilage. Following E12.5 tamoxifen treatment, LacZ positive descendants were localized more predominantly ventrally and were more restricted to the forming digit collateral ligaments and vinculum in some areas (connective tissue forming tendon sheaths) [17]. By E13.5 treatment, LacZ positive descendants were entirely limited to the collateral ligaments and vinculum. Hence, the strong whole mount LacZ staining around digit joints seen at later stages (Fig 3) derives from recombination in the ligaments around these joints (Fig 4). Notably, although Scleraxis-positive progenitors give rise to both tendons and ligaments [18], Bmp2CreER activity induced at various stages became restricted to the collateral ligaments by E15.5, with the exception of some mosaic LacZ positive descendants in the major ventral flexor tendon (flexor digitorium profundis) of digit 5. Consequently, Bmp2CreER activated at later stages (E12.5–13.5) may be useful for selectively labeling ligament progenitors and distinguishing ligament from tendon formation in the digit region.

**Fig 5:**
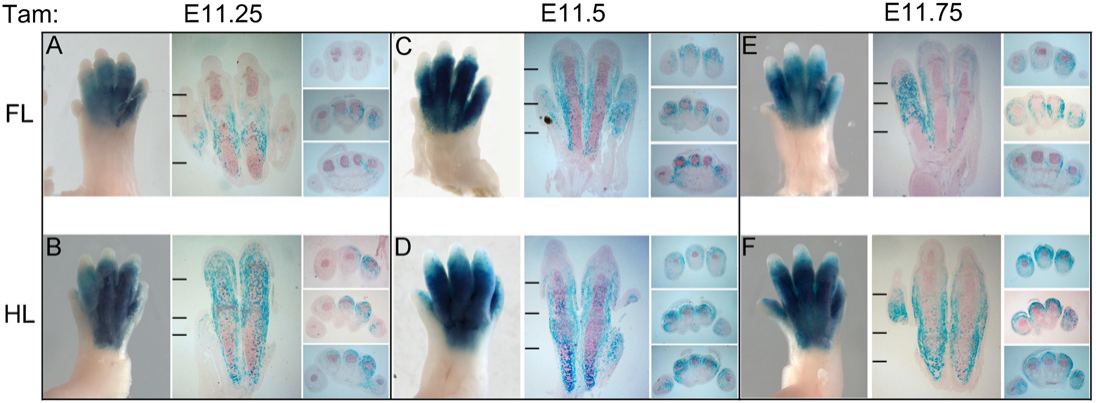
Comparison of Bmp2CreER activity levels and distribution assayed at E15.5 in LacZ-positive descendants, following tamoxifen (Tam) treatment between stages E11.25 to E11.75. For each tamoxifen (Tam) treatment time, whole mount and adjacent longitudinal sections through dorsoventral axis of forelimb (FL) or hindlimb (HL) are oriented with distal at top, and anterior-to-posterior (digit 1–5) from left-to-right. Hatch marks in panels of longitudinal sections show the levels at which cross-sections through digit region were taken (rightmost panel sets; distal to proximal). Cross-sections are all oriented with dorsal at top and anterior-to-posterior (digit 1–5) from left-to-right. Note that Cre activity transitions from digit condensations and interdigital mesenchyme to primarily interdigits and collateral ligaments during this time window of tamoxifen administration.

Interdigital mesenchyme has been proposed to play several roles at late stages of limb development. Because Bmp2CreER is expressed mainly in the interdigital mesenchyme and shows dynamic expression changes around E11.5, the distribution of LacZ positive descendants at E15.5 was also compared after single tamoxifen doses given from E11.25—E11.75 (Fig 5). As also seen in E13.5 descendants, E15.5 LacZ activity showed the broadest distribution following an E11.5 tamoxifen dose. Although there was some labeling of chondrogenic cells in the tips of digit condensations, the highest expression was present in immediately surrounding perichondrial and soft tissue. By E11.75, perichondrial staining began to decline and LacZ staining was already becoming more strongly localized to collateral ligaments (Fig 5E).

**Fig 6:**
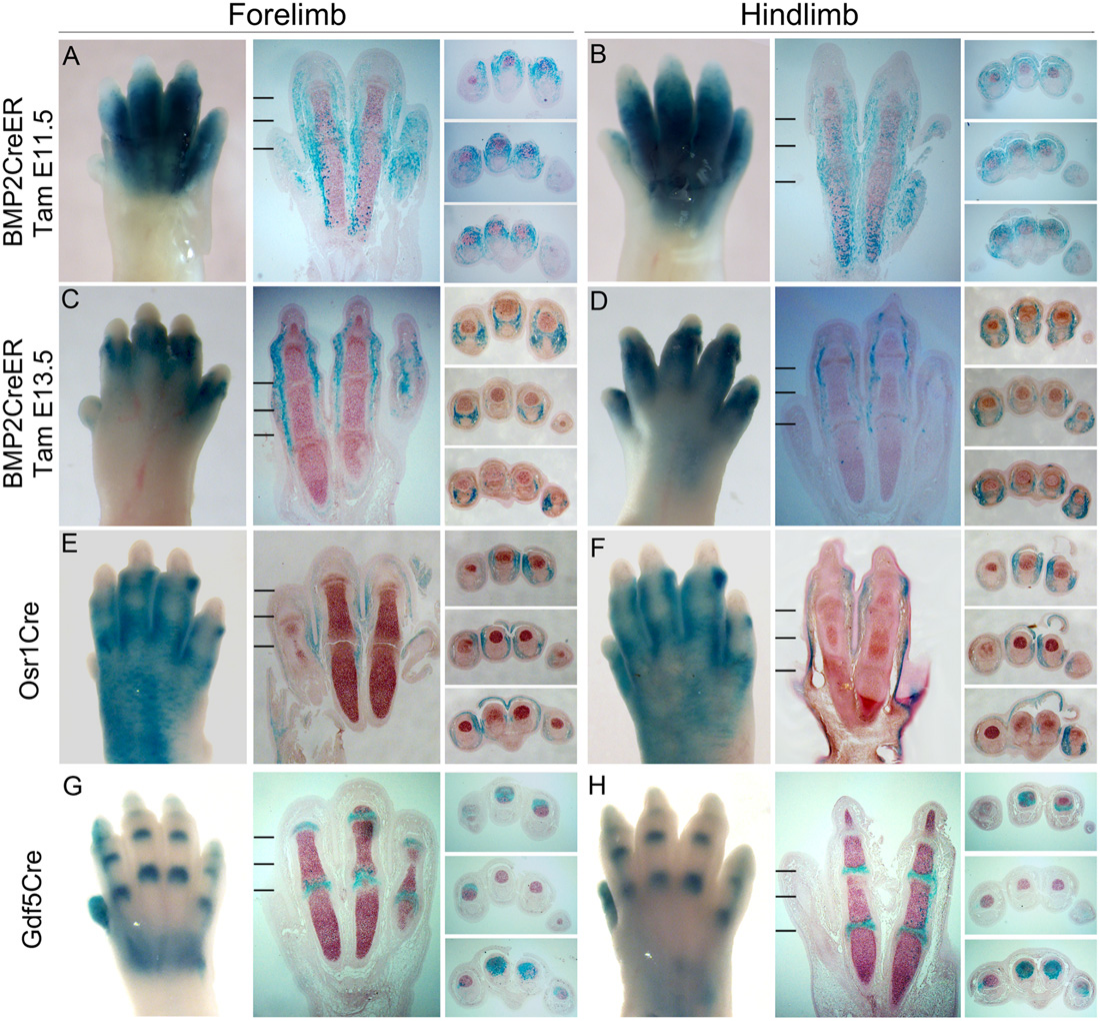
Bmp2CreER, Osr1Cre and Gdf5Cre have distinct and partly complementary distributions of recombinase activity. Bmp2CreER activity after single dose tamoxifen (Tam) administration at 2 different times (E11.5, A-B or E13.5, C-D) compared to Osr1Cre (E-F) and Gdf5Cre (G-H) in E15.5 limbs. In each case, whole mount and adjacent longitudinal sections through dorsoventral axis of forelimb (FL) or hindlimb (HL) are oriented with distal at top, and anterior-to-posterior (digit 1–5) from left-to-right. Hatch marks in panels of longitudinal sections show the levels at which cross-sections were taken (rightmost panel sets; distal to proximal). Cross-sections are all oriented with dorsal at top and anterior-to-posterior (digit 1–5) from left-to-right. Note that Bmp2CreER labels LacZ-positive descendants mainly in interdigital mesenchyme, including collateral and joint ligaments, whereas Osr1Cre descendent cells reside in subcutaneous tissue and joint ligaments, and Gdf5Cre labels cells lining the joint regions and immediately adjacent subarticular and perichondrial cells at E15.5.

The Bmp2CreER line described here has a distinct, and partly complementary expression pattern compared to other reported Cre lines that are active in the distal limb bud at similar developmental stages (see Fig 6). Previously, an Osr1Cre transgenic line that drives recombination in interdigits (~E13.5) [14] and a Gdf5Cre transgenic line that selectively labels the joint regions at later stages (~E14.5) [19, 20] have been described. Bmp2CreER activation at E11.5 can drive recombination in the interdigit tissue including the capsule surrounding the forming digit joints, but not within joint progenitors (interzone) or joint lining cells at E15.5. By E13.5, activity becomes limited mainly to collateral ligament progenitors. In contrast, although Osr1Cre is first expressed strong throughout the interdigital mesenchyme at a slightly later stage (by about E13.5), Osr1Cre-labeled LacZ positive cells were more peripherally distributed sub-epithelially, as well as in ligaments connecting joint regions. No LacZ positive cells were seen within in cartilage, or joint lining. On the other hand, Gdf5Cre-labeled LacZ positive cells were restricted almost exclusively to the joint lining and adjacent subarticular and perichondrial tissue.

## Conclusions

The Bmp2CreER line is expressed predominantly in the region of the forming digital plate and becomes mainly restricted to the interdigital region following the appearance of digital rays prior to the onset of programmed cell death in interdigits. Hence, this conditional Cre driver complements other Cre lines with late autopod expression, and may have utility in analysis of interdigit roles during limb development. After E12.5, Cre activation results in selective labeling of the major digit ligaments and may also provide a tool for dissecting the divergence of ligament from tendon lineages in the distal autopod.

## Materials and Methods

### Ethics Statement

All animal studies were approved by and carried out according to the Institutional Animal Care and Use Committee (IACUC) guidelines at NCI-Frederick, Frederick, MD under protocol number ASP-12-405.

### Transgenic constructs and generation of Transgenic mice

A 2.83kbp SfiI-SalI fragment containing the beta-globin intron fused to the CreER (T1) coding region derived from a pSG5 vector [21] was inserted into plasmid 1069_Bmp2Cre enhancer K1 [15] in the pSfi-Hsp68LacZ vector) that had been digested with EheI-SalI, replacing the LacZ coding region. The resulting 8.8kbp Bmp2CreER-T-SV40pA expression construct (AloI-SalI fragment) was injected into C57BL/6NCr zygotes to generate transgenic lines, as previously described by [22]. Embryos were transferred to foster mothers and recovered after birth for analysis and establishing lines. Transgenic founders were identified by Southern blot analysis using a CreER coding probe. For subsequent genotyping of animals in an established line, polymerase chain reaction primers from the Cre coding region were used (5’-GAAAATGCTTCTGTCCGTTTGC-3’ and 5’-ATTGCTGTCACTTGGTCGTGGC-3’; 207 bp amplicon).

The Rosa26LacZ reporter line [23] was used to assess recombinase activity by crossing to Bmp2CreER transgenic mice and analyzing LacZ activation in Bmp2CreER; Rosa26LacZ compound heterozygous embryos. The Osr1Cre [14] and Gdf5Cre [19] lines have been previously described, and were used in similar crosses for comparing embryonic Cre activity. Control progeny harvested either following injection with vehicle alone, or without IP injection of tamoxifen had little to no evidence of Cre activity (data not shown).

### Tamoxifen Injections and Embryo Analyses

For determination of embryonic ages, females were examined each morning for the presence of a post-coital plug, and noon on the day that a plug was observed was considered to be E0.5. As an independent confirmation that the developmental stage matched the plug age for embryos collected between ~E10.5—E13.5, the limb bud morphology was carefully examined and compared to published criteria [24]. Tamoxifen (Sigma, T-5648) was administered by intraperitoneal (IP) injection as previously described [13, 25]. Pregnant mice averaging about 25gm in body weight, were given a single IP dose of 3mg tamoxifen (10 mg/ml in vegetable oil), at times indicated in text ranging from E9.5 to E14.5. For analysis of LacZ activity, embryos were dissected into PBS and fixed with 4% paraformaldehyde and 0.2% glutaraldehyde in PBST (PBS with 0.1% Tween-20) at 4°C for 1 hr, then washed 3 times with PBST at room temperature. For analysis of E15.5 limbs, the skin was slit open at several sites prior to fixing. Whole-mount LacZ staining within X-Gal substrate was performed as described previously [25]. For histologic examination, embryos were stained for 18–24 hours and post-fixed for 1 hour in 4% PFA prior to embedding for paraffin sections [13].
